# Unpredicted but It Exists: Trigonal Sc_2_Ru with a Significant Metal–Metal Charge Transfer

**DOI:** 10.1021/acs.inorgchem.1c01168

**Published:** 2021-07-09

**Authors:** Riccardo Freccero, Pavlo Solokha, Serena De Negri

**Affiliations:** Dipartimento di Chimica e Chimica Industriale, Università degli Studi di Genova,via Dodecaneso 31, Genova 16146, Italy

## Abstract

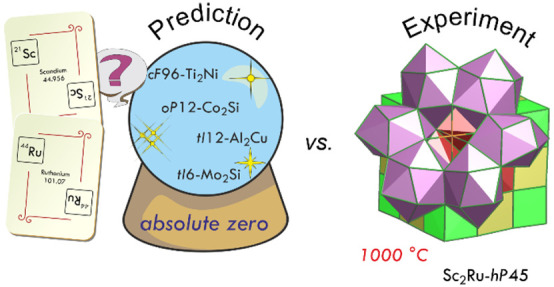

The Sc_2_Ru compound, obtained by high-temperature synthesis, was found to crystallize in a new trigonal *hP*45 structure type [space group *P*3̅*m*1; *a* = 9.3583(9) Å and *c* = 11.285(1) Å]: Ru@Sc_8_ cubes, Ru@Sc_12_ icosahedra, and uncommon Ru@Sc_10_ sphenocoronae are the building blocks of a unique motif tiling the whole crystal space. According to density functional theory studies, Sc_2_Ru is a metallic compound characterized by multicenter interactions: a significant charge transfer occurs from Sc to Ru, indicating an unexpectedly strong ionic character of the interactions between the two transition metals. Energy calculations support our experimental results in terms of stability of this compound, contributing to the recurrent discussion on the limits of the high-throughput first-principles calculations for metallic materials design.

## Abstract 2

A strong metal (Sc) to metal (Ru) charge transfer occurs for the new Sc2Ru compound, indicating a significant ionic contribution to the bond. The Sc_2_Ru trigonal crystal structure features a unique structural motif built up by Ru-centered simple cubes and icosahedra connected through hybrid sphenocorona polyhedra. This investigation represents also a little food for thought on the interplay between the experiments and theoretical prediction in metallic materials design.

The prediction and design of new materials with desired properties is one of the main trends in materials science. To this aim, the classical phenomenological trial-and-error approach is more and more supported by a theoretical one, based on first-principles energy calculations, which are able to produce and screen an huge volume of data on composition/structure/properties.

This route is particularly useful in the study of materials based on expensive elements and difficult to synthesize, for example, because of their high formation temperatures and the need for long equilibration times. This is the case of the alloys of the platinum group metals, which are of great practical interest in many fields, including catalysis and electronic and jewelry applications. From the structural chemical point of view, these compounds range from simple Laves and Hume–Rothery phases^1^ to complex approximants and quasicrystals.^2−4^ The stability of an impressive number of new phases in numeorus binary systems of transition elements (*T*) was predicted by Curtarolo et al.^5−7^ To do that, a big structural data set of artificially generated compounds was created and their calculated formation energies at 0 K were compared in terms of the convex hull construction. The collected outcomes, however, show some discrepancies with the available experimental data,^8^ including our recent results on the constitutional properties of several Sc–*T* systems.^2^

Here, we focus on the Sc_2_*T* family of compounds: the state-of-the-art on their existence and crystal structure is summarized in Figure 1 (the experimental data were taken from Pearson’s crystal data^8^ and the high-throughput data from the AFLOW library^7^). The Sc_2_*T* phases with *T* = Mn, Re, Fe, and Rh were neither obtained nor predicted, so that their existence should probably be excluded; indeed, the experimental and calculated results also completely agree for Pd, Pt, and Au. The other data are more controversial and deserve some additional comments.

**Figure 1:**
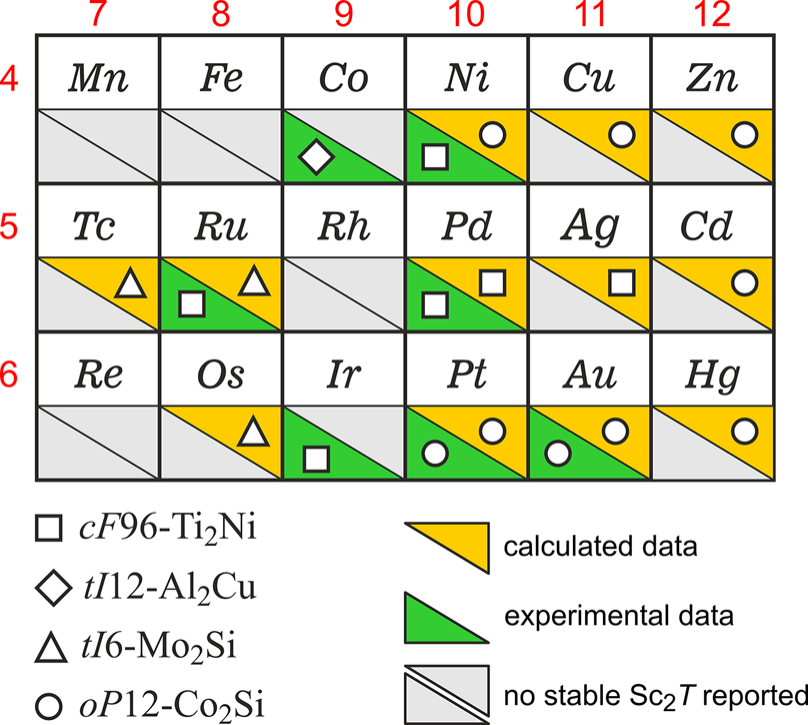
Comparison between the experimental and calculated literature data on Sc_2_*T* compounds (*T* = late transition metal).

The Sc_2_Co and Sc_2_Ir phases have been obtained experimentally, but they are unstable according to the density functional theory (DFT) calculations. Because the calculated results are only valid at low temperatures, the existence of the mentioned phases is very probable, taking also into account that their formation energies lie only slightly above the decomposition line of the convex hull. In many cases, i.e., for Os, Cu, Ag, Zn, Cd, Hg, the Sc_2_*T* compounds were only predicted to be stable at low temperatures but not experimentally confirmed. Finally, for Ni and Ru, a mismatch is observed between the experimental (*cF*96-Ti_2_Ni) and computed (*oP*12-Co_2_Si for Sc_2_Ni and *tI*6-Mo_2_Si for Sc_2_Ru) crystal structures.

This survey suggests that a deeper investigation is necessary to clarify the existence and structure of these intermetallics. Here, the Sc_2_Ru phase was targeted for a comparative investigation, from both the experimental and computational points of view. The Sc_2_Ru crystal structure, indeed, is not the only disagreement between the Sc–Ru intermetallics known from the literature^9,10^ and those calculated to be stable^7^ (see the Supporting Information, SI).

An almost single-phase sample of energy-dispersive X-ray spectroscopy (EDXS) measured composition 66.1 atom % Sc and 33.9 atom % Ru was obtained by direct synthesis in an arc furnace followed by annealing at 1000 °C for 7 days. The recorded powder X-ray diffraction (XRPD) pattern turned out to be incompatible with the published cubic Sc_2_Ru structure (*cF*96-Ti_2_Ni),^9^ which was suggested on the basis of a reduced number of reflections collected in a Debye focusing camera without any further structural refinement.

Thus, from a successful single-crystal investigation (see the SI), it was concluded that the synthesized Sc_2_Ru possesses trigonal symmetry (*P*3̅*m*1 space group) and shows an unprecedented spatial atom distribution. Its structural model contains seven Sc and five Ru independent crystallographic positions, corresponding to 30 Sc and 15 Ru atoms per cell (Table 1). The coordination numbers for each species were evaluated on the basis of the maximum gap rule.^11^

**Table 1:** Atomic Coordinates, Equivalent Isotropic Displacement Parameters (*U*_eq_), Coordination, and QTAIM Effective Charges (*Q*^eff^) for Each Species within the Sc_2_Ru Unit Cell

Atom	Site	*x*/*a*	*y*/*b*	*z*/*c*	*U*_eq_ (Å^2^)	Coordination	*Q*^eff^
Sc1	6*i*	0.12782(7)	*x̅*	0.15219(5)	0.0127(1)	15-Ru_5_Sc_10_	+1.16
Sc2	6*i*	0.17733(6)	*x̅*	0.43212(4)	0.0076(1)	14-Ru_6_Sc_8_	+1.23
Sc3	6*i*	0.49227(3)	*x̅*	0.26562(4)	0.0081(1)	13-Ru_5_Sc_8_	+1.20
Sc4	6*i*	0.79106(3)	*x̅*	0.09583(4)	0.0112(1)	12-Ru_4_Sc_8_	+1.06
Sc5	2*d*	^1^/_3_	^2^/_3_	0.11231(7)	0.0089(2)	13-Ru_4_Sc_9_	+1.08
Sc6	2*d*	^1^/_3_	^2^/_3_	0.60189(8)	0.0085(2)	13-Ru_7_Sc_6_	+1.34
Sc7	2*c*	0	0	0.35247(9)	0.0155(2)	14-Ru_4_Sc_10_	+0.96
Ru1	6*i*	0.83669(3)	*x̅*	0.32732(2)	0.0082(1)	12-Sc_10_Ru_2_	–2.35
Ru2	3*f*	^1^/_2_	0	^1^/_2_	0.0076(1)	14-Sc_8_Ru_6_	–1.56
Ru3	3*e*	^1^/_2_	0	0	0.0100(1)	12-Sc_12_	–2.88
Ru4	2*d*	^1^/_3_	^2^/_3_	0.34711(3)	0.0062(1)	11-Sc_8_Ru_3_	–2.18
Ru5	1*a*	0	0	0	0.0106(1)	6-Sc_6_	–2.88
Sc_2_Ru: *hP*45, own structure type, space group *P*3̅*m*1; *a* = 9.3583(9) Å, *c* = 11.285(1) Å							

As it comes out from interatomic distance analysis (see the SI), the coordination of Ru atoms reflects their tendency to maximize the number of heterocontacts at distances ranging from about 2.65 to 3.51 Å. Considering this, it seems convenient to represent the crystal structure as an assembly of Ru-centered polyhedra having exclusively Sc atoms at the vertices (Figure 2a). The simplest ones are almost regular cubes around the Ru2 and Ru4 sites; somewhat distorted icosahedra surround the Ru3 positions; sphenocorona polyhedra (CN = 10) coordinate the Ru1 positions. Sphenocorona could be viewed as a hybrid between the cube and icosahedron, being composed of 2 quadrangular and 12 triangular faces. Thanks to its shape properties, it is suitable for joining cubic and icosahedric moieties. In fact, a unique structural motif is discernible, which is composed of the above-described Ru-centered polyhedra in the following way: 19 Ru@Sc8 cubes form a Rubik’s cube-like skeleton, in the cavity of which six Ru@Sc10 sphenocoronae are embedded by means of their quadrangular faces. The triangular faces of sphenocoronae perfectly match two assemblies of six icosahedra, at the octahedral centers of which the Ru5 atoms sit. In summary, the structural motif is composed by a cubic topology core, along the diagonal of which (coincident with the *c* direction) the other fragments are joined together, maintaining the 3-fold rotational symmetry intrinsic for trigonal crystals. The whole Sc_2_Ru crystal space is built by a simple 3D tiling of this motif. Viewing the crystal structure along the *c* axis, a simple linear −ABAB– stacking of two types of slabs (A and B in Figure 2b) can be envisaged.

**Figure 2:**
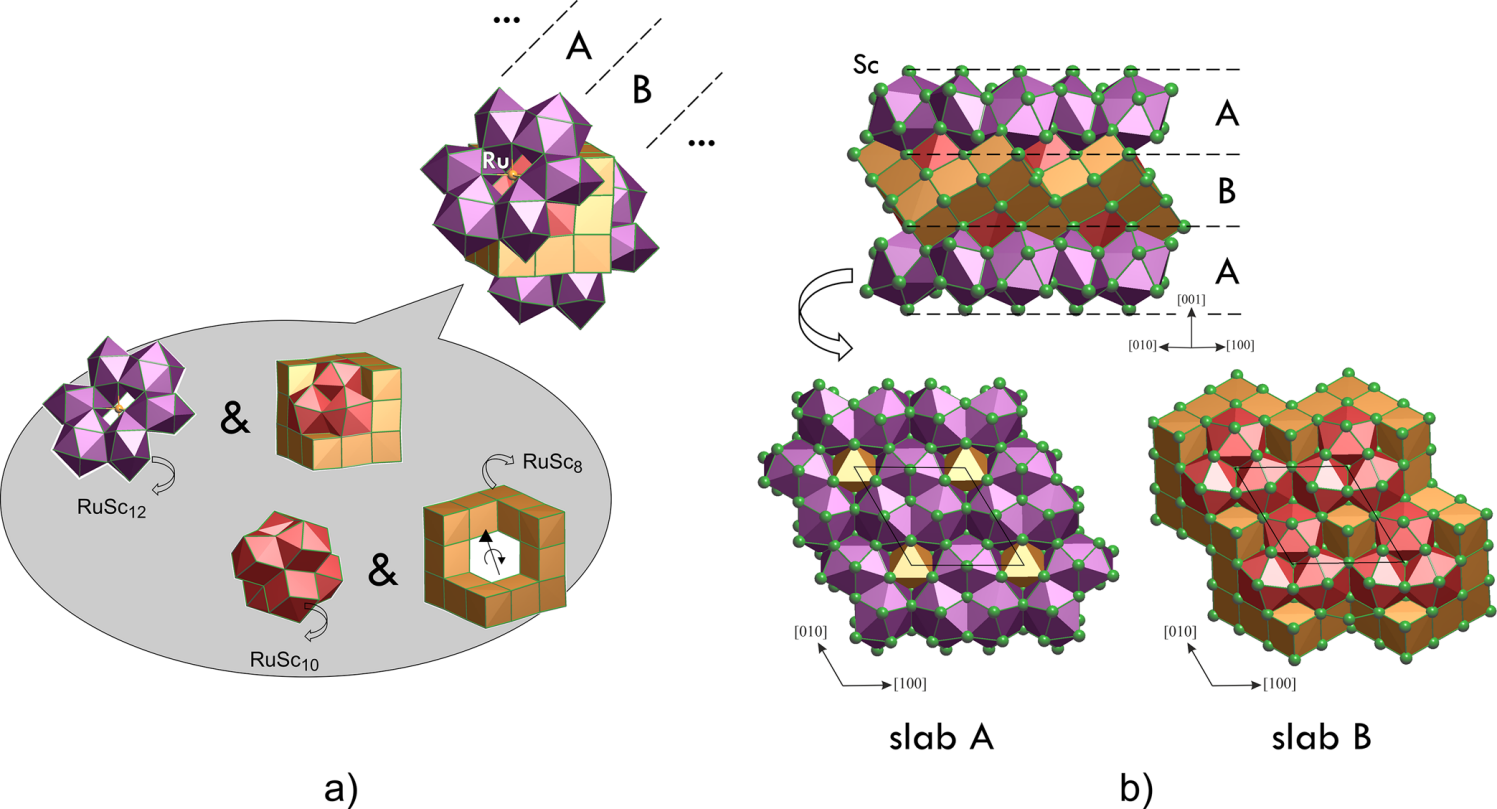
(a) Smallest building blocks of the Sc_2_Ru structure (Ru@Sc8 cubes, Ru@Sc12 distorted icosahedra, and Ru@Sc10 sphenocoronae) and their joint to form a unique structural motif with a 3-fold rotation symmetry. (b) Top: Crystal structure represented as a ABAB-mode linear intergrowth of two slabs built up from moieties depicted in part a. Bottom: A and B slabs viewed from a different perspective.

The effective charges (*Q*^eff^; Table 2) for each atomic species were obtained on the basis of the calculated electron density applying the QTAIM approach.^12^ The average charges of Sc and Ru are +1.15 and –2.30, respectively. Even though these values qualitatively agree with the electronegativities (1.19 for Sc and 1.54 for Ru in the Allen scale^13^), they clearly indicate a significant ionic contribution to the bond, larger than what would be typically expected for transition element intermetallics. Similar scenarios were already reported for some 2:1 compounds, like Al_2_Cu^14^ and Al_2_Pt,^15^ and other chemically related binaries;^16^ for Be_5_Pt, this charge separation was considered among the factors inducing the formation of a gap in the density of states (DOS).^17^ Atomic basins for each Ru species are represented in Figure 3 distributed within the aforementioned A and B slabs. They share convex surfaces with the surrounding Sc and, when they occur, flat ones with neighboring Ru basins. The charge of each Ru may be addressed by considering the number of surfaces it shares with Sc basins: in addition to the shortest heterocontacts (dark green Sc in Figure 3), Ru4 and Ru5 basins share surfaces also with further Sc (greenish in Figure 3). This explains, for instance, why the Ru4 charge (2.18) is higher than that of Ru2 (1.56).

**Table 2:** Experimental and Calculated Parameters as Well as Formation Enthalpies for Different Sc_2_Ru Models

	Experimental		Calculated				
Structural model	*hP*45-Sc_2_Ru	*cF*96-Ti_2_Ni^9^	*hP*45-Sc_2_Ru	*cF*96-Ti_2_Ni	*oP*12-Co_2_Si	*tI*12-Al_2_Cu	*tI*6-Mo_2_Si
*a* (Å)	9.358(1)	12.30	9.3591	12.2213	6.8137	6.3218	3.2348
*b* (Å)	9.358(1)	12.30	9.3591	12.2213	4.0617	6.3218	3.2348
*c* (Å)	11.285(1)	12.30	11.2557	12.2213	8.4153	5.9441	11.2916
*V* (Å^3^/f.u.)	57.1(2)	58.1	56.9	57.0	58.2	59.4	59.1
Δ_f_*H* (eV/atoms)			–0.449	–0.376	–0.398	–0.427	–0.461

**Figure 3:**
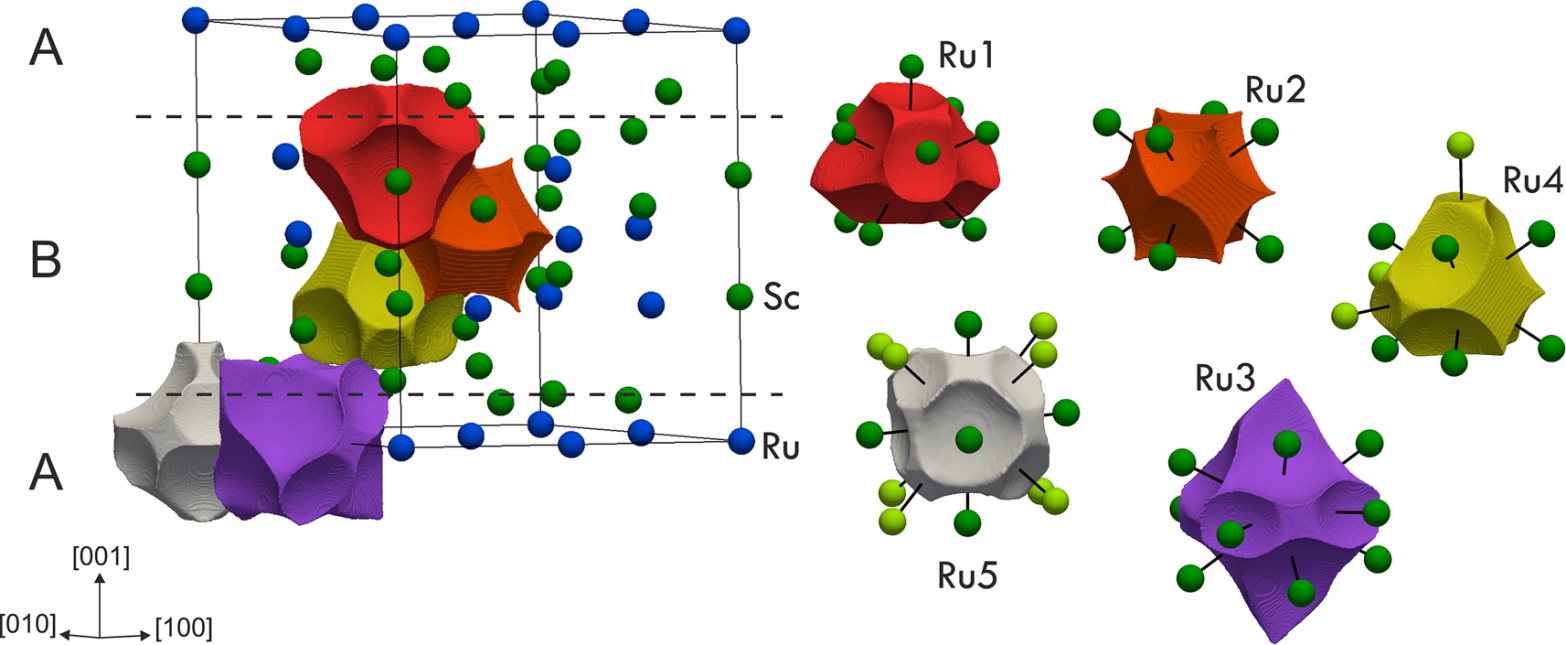
Shape of the QTAIM atomic basins for Ru species and their location in the Sc_2_Ru unit cell (left side). Ru basins are shown to the right, highlighting the surrounding Sc atoms whose QTAIM basins share a surface with the selected Ru. The Sc closest atoms (see the CN considered for the structural description) are shown in dark green and the others in greenish. The limits of the A and B slabs within the unit cell are indicated by dotted lines.

The electronic DOS reveals that Sc_2_Ru is a metallic phase (Figure 4).

**Figure 4:**
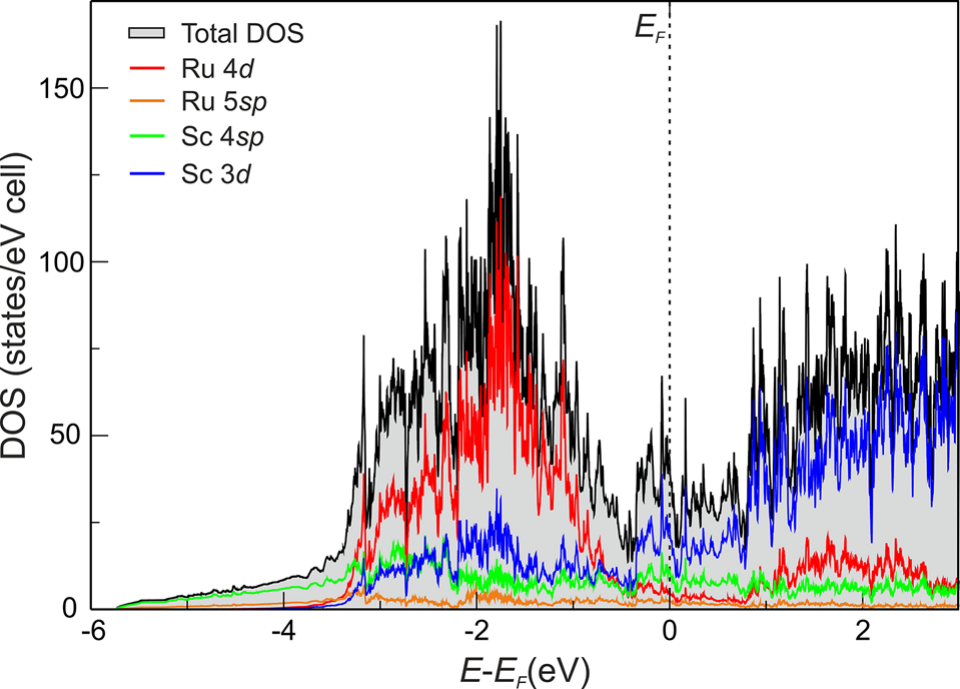
Total and projected electronic DOSs for Sc_2_Ru.

The presence of a pseudogap, although not particulary pronounced, indicates an electronic favorable scenario. The metallic behavior, together with the low valence electron concentration, suggests the presence of multicenter bonds. The energy window between −3.5 and −1 eV is primarily dominated by Ru 4*d* states, whereas the conduction band above *E*_F_ is mainly constituted by Sc 3*d*, suggesting a charge transfer in agreement with *Q*^eff^.^18,19^ Interestingly, Sc 3*d* contributes significantly to the valence region, even more than Ru, just below *E*_F_ from ca. −0.5 to 0 eV; their mixing with the Ru *d* states in a wide energy range also supports the presence of polar Sc–Ru interactions.

With the aim to corroborate our experimental results, DFT-based energy calculations have been performed with the *Quantum Espresso* software,^20^ considering the set of four structural models reported for Sc_2_*T* phases (Figure 1 and Table 2), complemented by the new *hP*45 prototype.

Formation enthalpies Δ_*f*_*H*obtained for different structural models on the basis of DFT (PBE) calculations using different codes show the same trend (Table S4 and Figure S6).

According to our results, at 0 K the most stable model is *tI*6-Mo_2_Si, closely followed by the title *hP*45-Sc_2_Ru, whose formation energy is higher by only 12 meV/atom, which is not that significant. The previously reported *cF*96-Ti_2_Ni is the last in the formation energy rank, being 73 meV/atom less stable than the new *hP*45 structure type, so that its existence could be excluded.

In conclusion, in this Communication, the new Sc_2_Ru compound, stable at 1000 °C, is presented from the crystal and electronic structure perspectives. Despite its expected metallic-like character featuring multicenter bonds, the significant charge transfer from Sc to Ru attests to a strong ionic character of the metal–metal interactions, quite unexpected considering that both are *d* elements.

The Sc_2_Ru structural peculiarity consists of a unique assembly of Ru-centered icosahedral fragments typical for Sc-rich complex intermetallics,^21^ like Sc_11_Ru_4_, Sc_57_Ru_13_, and Sc_44_Ru_7_, together with simple cubic blocks of CsCl topology, as present in ScRu. These motifs are perfectly joined by uncommon “hybrid” sphenocorona moieties, so that Sc_2_Ru can be viewed as a bridge between simple and complex scenarios.

Our results highlight some limits of the materials design by a first-principles calculation approach: (1) crystal-chemical descriptors are not applied when constructing the configurational spaces for energy screening; (2) predictions are mainly performed on the basis of 0 K calculations, assuming the entropic contribution to be small at higher temperatures; (3) other factors like kinetics and formation mechanisms are not considered at all.

Missing such points, the experimental work remains the only way to discover materials with new structures and disclose the temperature effects. On the other hand, no prediction based on energy calculations could ensure that a material with a given structure is achievable, which can only be attained by synthetic efforts.

## Supporting Information Available

Comparative analysis of experimental and high-throughput data on the Sc–Ru binary compounds, synthesis, scanning electron microscopy and EDXS characterization of the obtained alloy, details on the X-ray studies and crystal structure solution accompanied by reconstructed intensity profiles, interatomic distances list, XRPD spectra, etc., and a detailed description of the computational techniques and results on formation energy trends (PDF)

## Accession Codes

CCDC 2071469 contains the supplementary crystallographic data for this paper. These data can be obtained free of charge via www.ccdc.cam.ac.uk/data_request/cif, or by emailing data_request@ccdc.cam.ac.uk, or by contacting The Cambridge Crystallographic Data Centre, 12 Union Road, Cambridge CB2 1EZ, UK; fax: +44 1223 336033.
