# Supplementary material for: Unpredicted but It Exists: Trigonal Sc2Ru with a Significant Metal–Metal Charge Transfer
Source: Inorg Chem. 2021 Jul 9;60(14):10084–8. doi: 10.1021/acs.inorgchem.1c01168 (PMC8389775; doi:10.1021/acs.inorgchem.1c01168)
Supplement: Supplementary file 1 — ic1c01168_si_001.pdf [file ic1c01168_si_001.pdf]

Supporting Information

Unpredicted but it exists: trigonal Sc_2Ru with a significant metal-metal charge transfer

Riccardo Freccero, Pavlo Solokha*, Serena De Negri

Dipartimento di Chimica e Chimica Industriale, Università degli Studi di Genova,
via Dodecaneso 31, 16146 Genova, Italy

Supporting information

Table of contents

Experiment vs. high-throughput data. S-2
Synthesis and SEM-EDXS characterisation S-3
X-ray studies and crystal structure solution S-3
Computational details and results S-7

Experiment vs. high-throughput data.

A comparison between experimental data and high-throughput first principles calculation results is illustrated in Figure S1 for the Sc-Ru system. In general, there is a good correlation between them except the cases indicated by red numbers for which, according to high-throughput data:

1) the Sc_5Ru_3 is unstable at low temperatures;
2) the experimentally obtained Sc_2Ru (*hP*96-Ti_2Ni) is far away from the decomposition line: there are several 2:1 stoichiometry structural models (including the ground state *tI*6-Mo_2Si) that possess noticeably lower formation energies;
3) the $Sc_{57}Ru_{13}$ is slightly unstable at low temperatures.

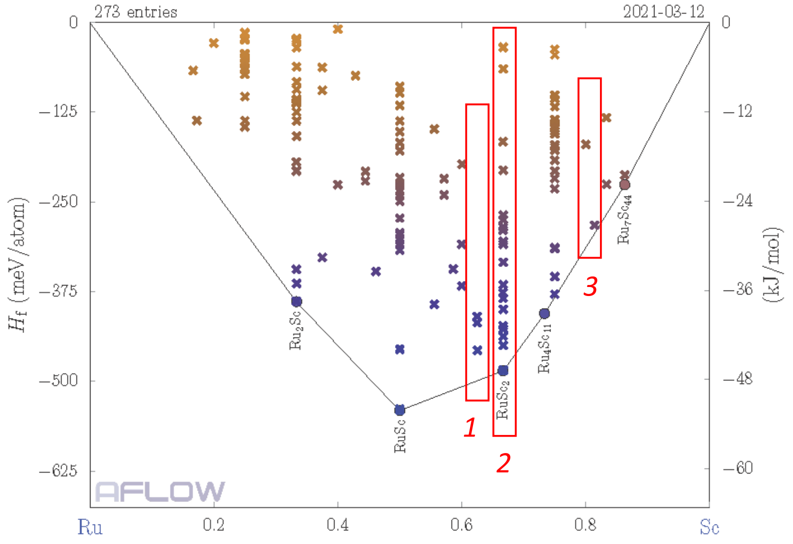

Figure S1. Sc-Ru convex-hull taken from http://www.aflow.org/aflow-chull implemented within the opensource, ab initio framework http://www.aflow.org. Red markers indicate discrepancies between experimental and high-throughput data.

Synthesis and SEM-EDXS characterisation

Stoichiometric amounts of Ru and Sc, with nominal purities > 99.9 mass% were used for synthesis of an alloy of *ca*. 0.8 g. The sample was arc melted several times under Ar atmosphere on a water-cooled copper hearth with a tungsten electrode; weight losses were less than 1 mass%. Subsequently, the obtained alloy was put in an alumina crucible, sealed in a silica tube under Ar atmosphere and heat treated at 1000 °C for 7 days. Annealing was followed by water quenching.

In order to obtain smooth surfaces suitable for microscopic analysis, some specimen fragments were embedded in a phenolic hot mounting resin with carbon filler, employing an automatic hot compression mounting press Opal 410 (ATM GmbH, Germany). The grinding procedure with abrasive SiC papers (grain size from 600 to 1200) was followed by diamond pastes (6, 3 and 1 μm) polishing. Microstructure examination (see Figure S2) and phase composition analyses were performed by a scanning electron microscope (SEM) Zeiss Evo 40 equipped with an Energy Dispersive X-ray (EDX) Spectroscope Oxford Instruments INCA X-ACT operated by the INCA Energy software (Oxford Instruments, Analytical Ltd., Bucks, U.K.). Calibration for the elemental analysis was performed on a cobalt standard.

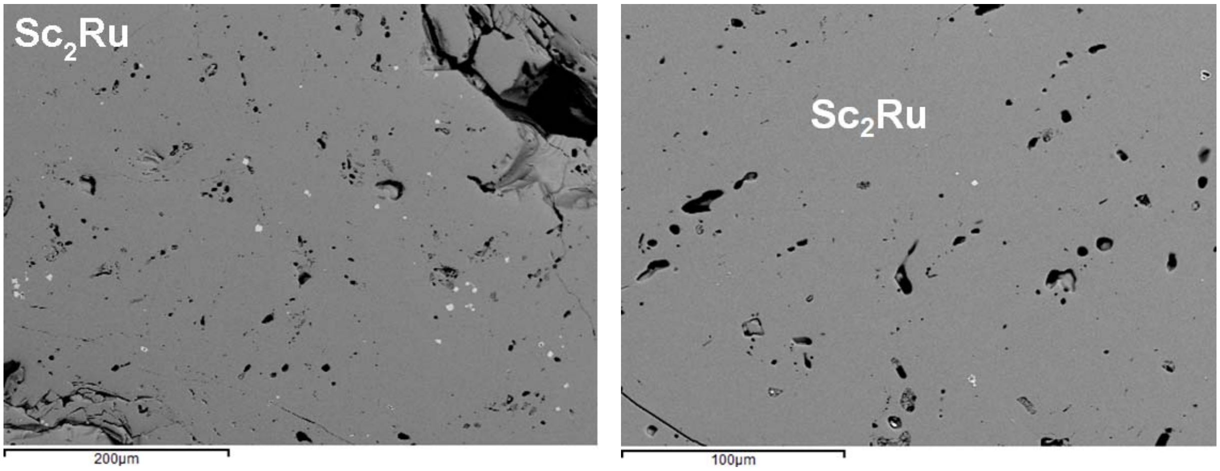

Figure S2. Different magnification micrographs (SEM-BSE mode) of the Sc_2Ru sample annealed at 1000 °C.

X-ray studies and crystal structure solution

The single crystal selection was conducted on the mechanically fragmented alloy. X-ray diffraction measurements were performed by a Bruker Kappa APEXII CCD area-detector diffractometer, using Mo K_α (λ = 0.71073 Å) radiation. Datasets were collected operating in ω-scan mode over the reciprocal space up to $\theta \approx 30°$, with an exposure time of 30 seconds per frame. The crystal-to-detector distance was fixed to 5 cm. Data integration, Lorentz polarization, and semiempirical absorption corrections were applied to all data by using the SAINT and SADABS softwares.[1] Crystal structure refinement was carried out by full-matrix least-squares methods on $|F|^2$ using the SHELXL program[2] as implemented in WinGX[3]. The indexation of collected intensity data yielded

to a trigonal symmetry unit cell with *a*=9.35 Å and *c*=11.29 Å (precession images of *h*0*l* and *hk*0 zones are shown in Fig. S3).

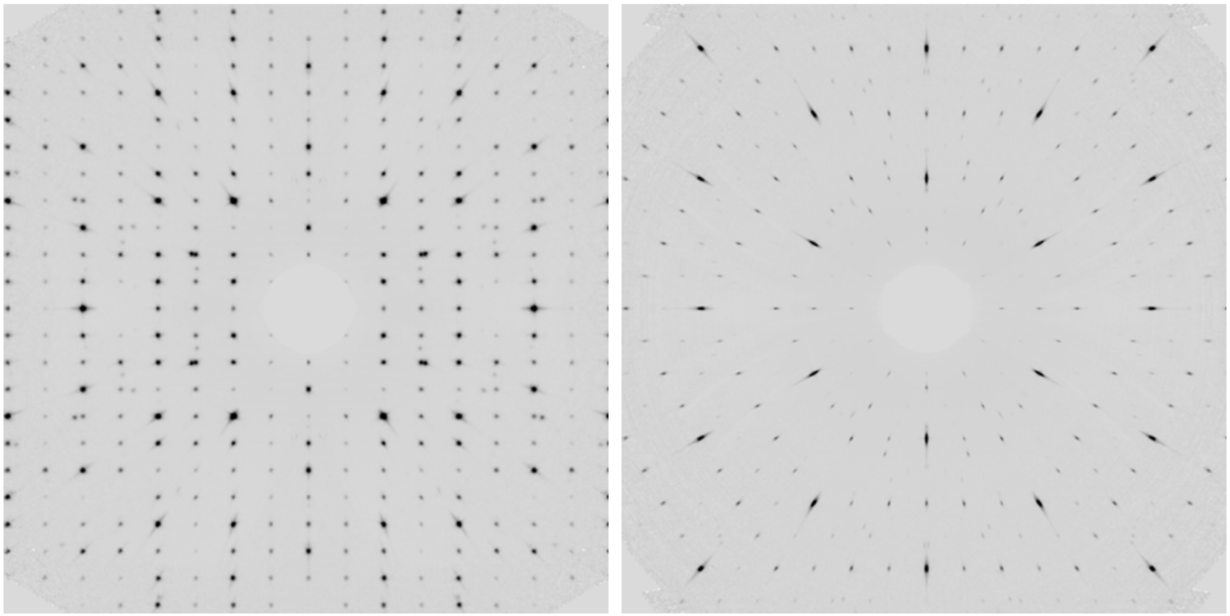

a) b)

Figure S3. Reconstructed intensity profiles for *h*0*l* (a) and *hk*0 (b) zones of the Sc_2Ru diffraction pattern.

Since no systematic absences were found, the following space-groups were suggested by XPREP[1]: *P*3 (143), *P*–3 (147), *P*321 (150), *P*3*m*1 (156), *P*–3m1 (164). The lowest combined figure of merit was associated to the last one. Indeed, the crystal structure was quickly solved in this space-group by means of the charge-flipping algorithm implemented in JANA2006[4] figuring out the almost complete structural model with 45 atoms per cell (5 Ru and 7 Sc Wyckoff sites). Site occupancy factors (SOF) and possible statistical mixture were carefully checked for each species in separate refinement cycles, but no significant deviation from an ordered scenario was detected, suggesting Sc_2Ru to be a stoichiometric compound. This is also in perfect agreement with the measured EDXS composition. A further refinement including anisotropic thermal displacement parameters resulted in acceptable residuals and flat difference Fourier map (Table S1). The generated CIF file has been deposited at Cambridge Crystallographic Data Centre (code-2071469). Details of the data collection and refinement are listed in Table S1. Interatomic distances for Sc_2Ru (*hP*45) - in Table S2 and constructed histograms are shown in Figure S4.

Table S1. Crystallographic data for the Sc_2Ru single crystal together with some experimental details on its structure determination.

Empirical Formula	Sc_2Ru	Calc. density [g·cm^{-3}]	5.56
EDXS composition	$Sc_{65.9}Ru_{34.1}$	Abs. coeff. [μ, mm^{-1}]	11.85
Depositing CSD-code	2071469	Unique reflections	1096
Structure type	Sc_2Ru	Reflections ($I > 2\sigma(I)$)	1010
Space group	$P\bar{3}m1$	Data/parameters	1096/54
Pearson symbol, Z	*hP*45, 15	*GOF*	1.165
Unit cell dimensions:		R_{int}/R_{sigma}	0.0223/0.0089
a [Å]	9.3583(9)	R_1/wR_1 ($I > 2\sigma(I)$)	0.0134/0.0330
c [Å]	11.285(1)	R_1/wR_1 (all data)	0.0163/0.0346
V [Å^3]	855.9(2)	$\Delta\rho_{fin}$ (max/min) [e/Å^3]	0.93/-1.18

Table S2. Interatomic distances (< 4.00 Å) for Sc_2Ru.

Central atom	Adjacent atoms	*d* (Å)	Central atom	Adjacent atoms	*d* (Å)	Central atom	Adjacent atoms	*d* (Å)
**Sc1**	Ru5	2.6911(6)	**Sc4**	Ru3 (2x)	2.6619(3)	**Ru1**	Sc7	2.6623(3)
	Sc4 (2x)	3.0248(5)		Ru1	2.7150(5)		Sc4	2.7150(5)
	Sc7	3.0660(1)		Sc1 (2x)	3.0248(8)		Sc2	2.7242(5)
	Sc4	3.0924(8)		Sc1	3.0924(8)		Sc6	2.8694(4)
	Ru1 (2x)	3.1178(4)		Sc5	3.0956(8)		Sc3 (2x)	2.8775(3)
	Sc2	3.2594(7)		Sc3 (2x)	3.1667(5)		Sc2 (2x)	3.0098(3)
	Sc3 (2x)	3.2849(7)		Sc4 (2x)	3.4925(9)		Sc1 (2x)	3.1178(7)
	Sc5	3.3615(6)		Ru5	3.5551(6)		Ru2 (2x)	3.3534(2)
	Ru3 (2x)	3.5136(6)		Sc6	3.9626(9)	**Ru2**	Sc3 (2x)	2.6479(5)
	Sc1 (2x)	3.5884(6)	**Sc5**	Ru4	2.6497(9)		Sc2 (4x)	2.7290(5)
	Ru4	3.9920(6)		Ru3 (3x)	2.9840(3)		Sc6 (2x)	2.9360(4)
**Sc2**	Ru4	2.7044(5)		Sc4 (3x)	3.0956(8)		Ru4 (2x)	3.2055(2)
	Ru1	2.7242(5)		Sc3 (3x)	3.1032(5)		Ru1 (4x)	3.3534(3)
	Ru2 (2x)	2.7290(3)		Sc1 (3x)	3.3615(6)	**Ru3**	Sc4 (4x)	2.6619(3)
	Ru1 (2x)	3.0098(6)	**Sc6**	Ru1 (3x)	2.8694(4)		Sc5 (2x)	2.9840(3)
	Sc7	3.0117(6)		Ru4	2.8752(9)		Sc3 (2x)	3.0001(5)
	Sc3 (2x)	3.1697(5)		Ru2 (3x)	2.9360(4)		Sc1 (4x)	3.5136(6)
	Sc6	3.1724(7)		Sc2 (3x)	3.1724(7)	**Ru4**	Sc5	2.6497(9)
	Sc2	3.2572(7)		Sc3 (3x)	3.1979(5)		Sc2 (3x)	2.7044(5)
	Sc2	3.2573(4)		Sc4 (3x)	3.9626(9)		Sc3 (3x)	2.7354(3)
	Sc1	3.2594(7)	**Sc7**	Ru1 (3x)	2.6623(2)		Sc6	2.8752(9)
	Sc7	3.7645(8)		Sc2 (3x)	3.0117(4)		Ru2 (3x)	3.2055(2)
**Sc3**	Ru2	2.6479(5)		Sc1 (3x)	3.0660(1)		Sc1 (3x)	3.9920(6)
	Ru4	2.7354(4)		Sc7	3.330(1)	**Ru5**	Sc1 (6x)	2.6911(4)
	Ru1 (2x)	2.8775(3)		Sc2(3x)	3.7645(8)		Sc4 (6x)	3.5551(6)
	Ru3	3.0001(5)		Ru5	3.9776(9)		Sc7 (2x)	3.9776(9)
	Sc5	3.1032(6)						
	Sc4 (2x)	3.1667(7)						
	Sc2 (2x)	3.1697(6)						
	Sc6	3.1979(6)						
	Sc1 (2x)	3.2849(4)						

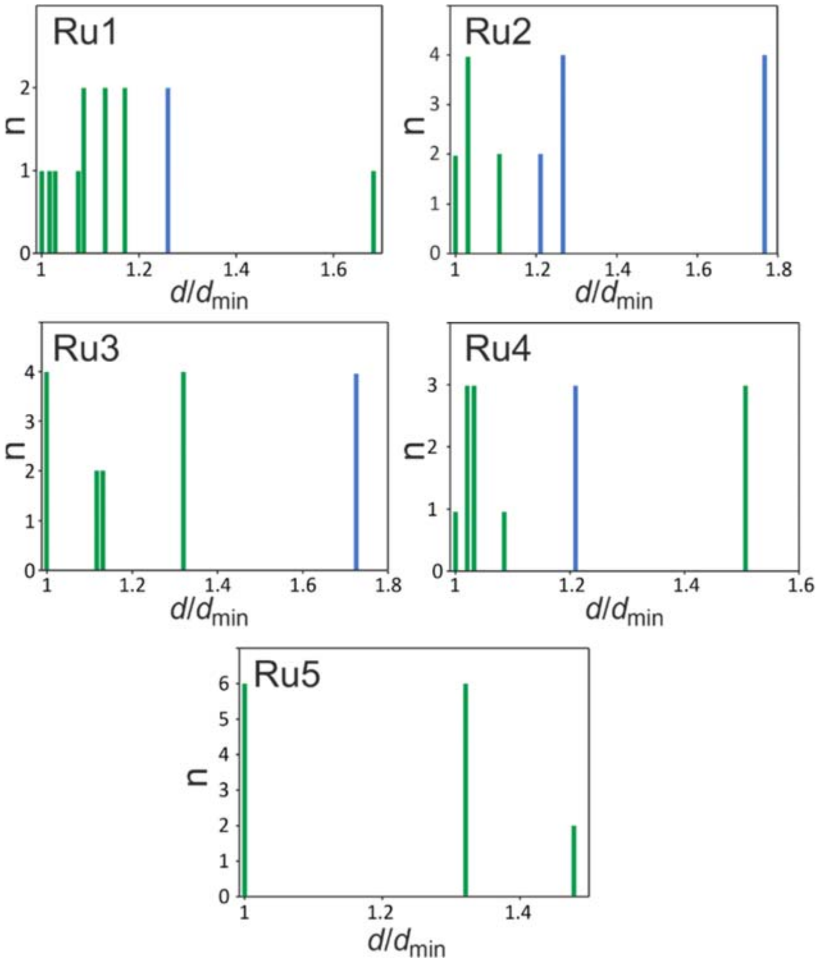

Figure S4. Number of atoms (n) surrounding the Ru species as a function of the reduced distance d/d_{min}. Green and blue bars indicate Sc and Ru, respectively.

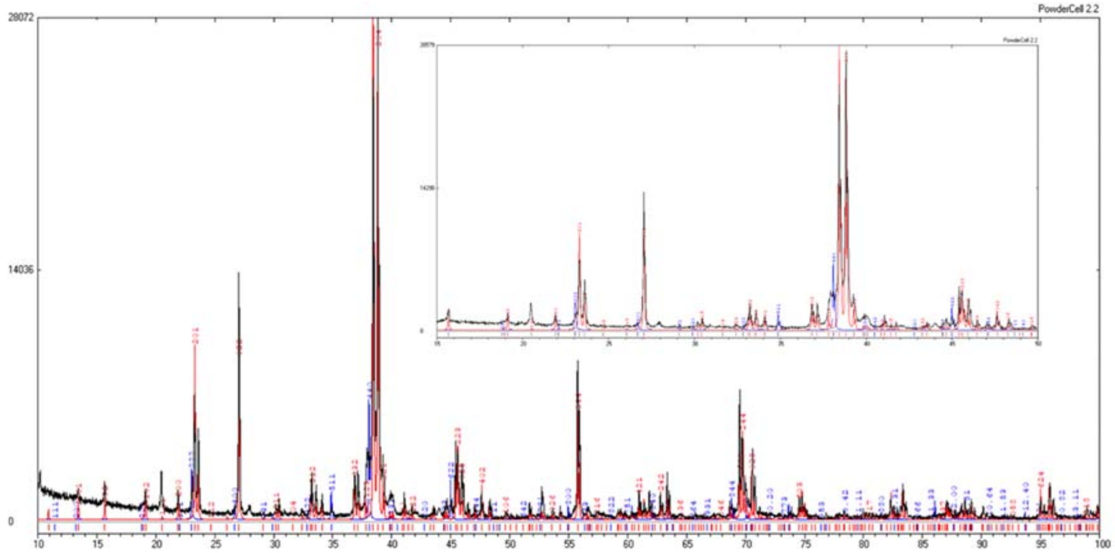

Figure S5. Comparison between experimental (black line) and theoretical diffraction patterns: Sc_2Ru (red line) and $Sc_{11}Ru_4$ (blue line, minor quantity). Inset shows a zoom of the 15–50° range of the pattern.

The X-ray powder diffraction pattern was collected using a Philips X'Pert MPD (Cu K_α radiation, scanning step *ca.* 0.015°) diffractometer; indexing was performed by Powder Cell software[5] (see Figure S5).

Computational details and results

DFT total energy calculations have been performed for different Sc_2Ru structural models, relaxed at the equilibrium geometry (in P1 space group) using both the plane wave pseudopotential software QUANTUM-ESPRESSO (QE)[6] and the all-electron, full-potential FHI-aims package[7,8]. In both cases, the PBE functional for the exchange and correlation energy was applied. Details on *k*-point mesh chosen to sample the Brillouin zone are reported in Table S3.

Table S3. Used *k*-point meshes for elemental Sc, Ru and Sc_2Ru intermetallic with different structures.

Phase	Structure	*k*-point mesh
Sc	*hP*2-Mg	18 18 12
Sc_2Ru	*hP*45-Sc_2Ru	6 6 4
	*cF*96-Ti_2Ni	6 6 6*
	*oP*12-Co_2Si	12 14 6
	*tI*12-$CuAl_2$	10 10 10
	*tI*6-Mo_2Si	18 18 4
Ru	*hP*2-Mg	20 20 14

*calculation performed for the primitive unit cell.

The QE calculations were conducted with the recommended projector-augmented wave (PAW)[9] pseudopotentials, available at the PSLI library[10]. The semicore 3*s* and 3*p* states for Sc and the 4*s* and 4*p* for Ru were treated as valence electrons. The plane-wave and density cut-off were set to 56 Ry and 540 Ry, respectively. The orbital occupancies at the Fermi level were treated with a Gaussian smearing of 0.01 Ry. In order to make a comparative analysis of our results with those available at the AFLOW repository, the QE obtained energies have been discussed in the main text for consistency since both QE and VASP are DFT codes based on pseudopotentials. The FHI-aims code was also then used to check the reproducibility of the results. For this purpose, predefined default "tight" basis sets for Sc and Ru were selected including scalar-relativistic effect according to the ZORA approximation. A Gaussian smearing of 0.01 eV was set.

The formation enthalpies ($\Delta_f H$) were evaluated using the calculated total-energies per atom (E), according to the following formula: $\Delta_f H = E(Sc_2Ru) - [\frac{2}{3}E(\mathrm{Sc}) + \frac{1}{3}E(Ru)]$.

These values are listed in Table S4 and shown in Figure S6, together with those available at the AFLOW library calculated with VASP code.

It has to be noted that, though the values of $\Delta_f H$ slightly differ, the energy differences between the already reported structural models obtained by G.L.W. Hart *et al.*[11] are very well reproduced with the computational procedures applied in this work.

Table S4. Formation enthalpies $\Delta_f H$ obtained for different structural models on the basis of DFT (PBE) calculations using different codes.

	$\Delta_f H$ (eV/at)				
Structural model	*hP*45 Sc_2Ru	*cF*96 Ti_2Ni	*oP*12 Co_2Si	*tI*12 Al_2Cu	*tI*6 Mo_2Si
VASP-aflow[12]	–	-0.400	-0.427	-0.450	-0.485
QE	-0.449	-0.376	-0.398	-0.427	-0.461
FHI-aims	-0.456	-0.384	-0.409	-0.434	-0.470

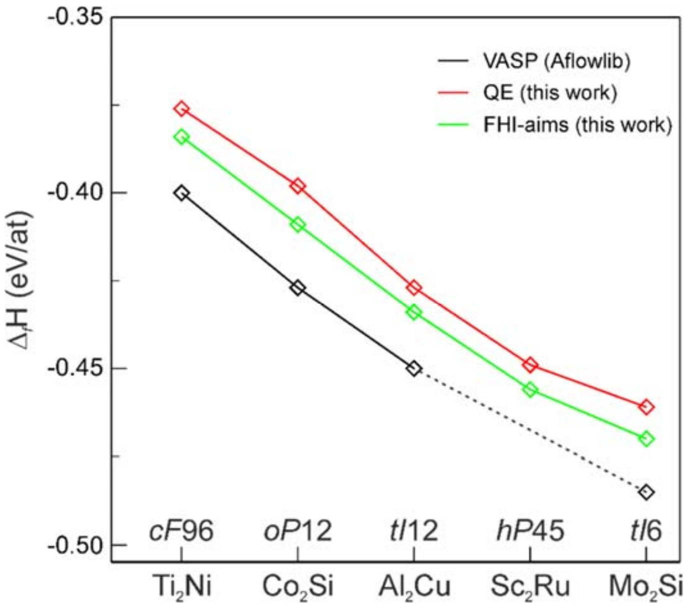

Figure S6. Formation enthalpies $\Delta_f H$ plot highlighting similar trends obtained employing different codes.

Finally, using the optimized Sc_2Ru–*hP*45 structure the total electron density (ED) was calculated on an equidistant grid of about 0.05 Bohr with the FPLO software[13], using an implemented module[14]. For this calculation, the Brillouin zone was sampled with a $10 \times 10 \times 10$ *k*-point mesh employing again the PBE exchange and correlation potential. The atomic basins and their average electronic populations were calculated by means of the DGrid software[15] on the basis of the Bader's Quantum Theory of Atoms In Molecules (QTAIM)[16]. To get the effective charges ($Q^{eff.}$), atomic populations were subtracted from the atomic numbers. QTAIM basins were visualized thanks to specific plug-ins for ParaView[17].

REFERENCES

(1) *Bruker. APEX2, SAINT-Plus, XPREP, SADABS, CELL_NOW and TWINABS*; Inc., Bruker AXS: Madison, WI, USA, 2014.

(2) Sheldrick, G. M. A Short History of SHELX. *Acta Crystallogr. Sect. A Found. Crystallogr.* **2008**, *64* (1), 112–122. https://doi.org/10.1107/S0108767307043930.

(3) Farrugia, L. J.; IUCr. WinGX and ORTEP for Windows : An Update. *J. Appl. Crystallogr.* **2012**, *45* (4), 849–854. https://doi.org/10.1107/S0021889812029111.

(4) Petříček, V.; Dušek, M.; Palatinus, L. Crystallographic Computing System JANA2006: General Features. *Zeitschrift für Krist. - Cryst. Mater.* **2014**, *229* (5). https://doi.org/10.1515/zkri-2014-1737.

(5) Kraus, W.; Nolze, G. POWDER CELL - A Program for the Representation and Manipulation of Crystal Structures and Calculation of the Resulting X-Ray Powder Patterns. *J. Appl. Crystallogr.* **1996**, *29* (3), 301–303. https://doi.org/10.1107/S0021889895014920.

(6) Giannozzi, P.; Baroni, S.; Bonini, N.; Calandra, M.; Car, R.; Cavazzoni, C.; Ceresoli, D.; Chiarotti, G. L.; Cococcioni, M.; Dabo, I.; et al. QUANTUM ESPRESSO: A Modular and Open-Source Software Project for Quantum Simulations of Materials. *J. Phys. Condens. Matter* **2009**, *21* (39), 395502. https://doi.org/10.1088/0953-8984/21/39/395502.

(7) Blum, V.; Gehrke, R.; Hanke, F.; Havu, P.; Havu, V.; Ren, X.; Reuter, K.; Scheffler, M. Ab Initio Molecular Simulations with Numeric Atom-Centered Orbitals. *Comput. Phys. Commun.* **2009**, *180* (11), 2175–2196. https://doi.org/10.1016/j.cpc.2009.06.022.

(8) Knuth, F.; Carbogno, C.; Atalla, V.; Blum, V.; Scheffler, M. All-Electron Formalism for Total Energy Strain Derivatives and Stress Tensor Components for Numeric Atom-Centered Orbitals. *Comput. Phys. Commun.* **2015**, *190*, 33–50. https://doi.org/10.1016/j.cpc.2015.01.003.

(9) Blöchl, P. E. Projector Augmented-Wave Method. *Phys. Rev. B* **1994**, *50* (24), 17953–17979. https://doi.org/10.1103/PhysRevB.50.17953.

(10) Kucukbenli, E.; Monni, M.; Adetunji, B. I.; Ge, X.; Adebayo, G. A.; Marzari, N.; de Gironcoli, S.; Corso, A. D. Projector Augmented-Wave and All-Electron Calculations across the Periodic Table: A Comparison of Structural and Energetic Properties. **2014**.

(11) Hart, G. L. W.; Curtarolo, S.; Massalski, T. B.; Levy, O. Comprehensive Search for New Phases and Compounds in Binary Alloy Systems Based on Platinum-Group Metals, Using a Computational First-Principles Approach. *Phys. Rev. X* **2014**, *3* (4), 041035. https://doi.org/10.1103/PhysRevX.3.041035.

(12) http://www.aflowlib.org/.

(13) Klaus, K.; Eschrig, H. Full-Potential Nonorthogonal Local-Orbital Minimum-Basis Band-Structure Scheme. *Phys. Rev. B - Condens. Matter Mater. Phys.* **1999**, *59* (3), 1743–1757.

https://doi.org/10.1103/PhysRevB.59.1743.

(14) Ormeci, A.; Rosner, H.; Wagner, F. R.; Kohout, M.; Grin, Y. Electron Localization Function in Full-Potential Representation for Crystalline Materials. *J. Phys. Chem. A* **2006**, *110* (3), 1100–1105. https://doi.org/10.1021/jp054727r.

(15) M. Kohout. DGrid, Dresden, v.5.0. 2018.

(16) Bader, R. F. W. *Atoms in Molecules : A Quantum Theory*; Clarendon Press, 1990.

(17) Ayachit, U. *The ParaView Guide: A Parallel Visualization Application*; 2015.
